# Supplementary material for: Dietary diversity and associated factors among women of reproductive age in Jeldu District, West Shoa Zone, Oromia Ethiopia
Source: PLoS One. 2022 Dec 19;17(12):e0279223. doi: 10.1371/journal.pone.0279223 (PMC9762567; doi:10.1371/journal.pone.0279223)
Supplement: Attachment — Submitted filename: Review Report.docx [file pone.0279223.s002.docx]

I would like to thank the authors for the nice work entitled “**Dietary Diversity and Associated Factors among Reproductive Age Women in Jeldu District, West Shoa Zone, Oromia Ethiopia**” aimed to assess dietary diversity and associated factors among reproductive age women. The clarity in the methodology and the result section were interesting. I have listed my concerns as follows:

1. Methods L 115- Here you have described to have used a tool after reviewing different document, can you please include the references including for the FAO document.
2. Methods L 115- You have mentioned the data collection tool is translated, can you please be specific with the translation process, if it’s translated by a certified language expert.
3. Methods L 123- You have stated that you used a 24-h recall method to assess the food consumed but you have not mention for how many days you implemented the technique. Please include the number of days.
4. Results L140- You have mentioned the average income of the participants to be about between 500-1500 birr. In my opinion it would sound better if you put this amount in USD, for ease of understanding to the readers. In what category of income is this amount considered in the Ethiopian context? Is it a Low, Middle or High income?
5. Results L 149-50- You have included the results for Women’s making decision, Agro ecological zone, having large livestock, having radio and mobile phone. Yet, in the methods section you have not described the justification for choosing these factors are considered appropriate for measuring/determining/ dietary diversity. Please provide your justification for including these factors instead of other possible options.
6. Result L150- You have used terms like ‘large livestock’ is large referring to number or size of the animals. Please define terms before using them.
7. Discussion L161- Your justification for adequate DD is the study period. Primarily, study period is a vague phrase is it the season or the length of your study duration? Secondly how about the effect of other factors such as access to the market, availability of food varieties in the area, tradition of the society under study, the feeding practices and trends etc.? Please try to consider the possible social, economic or environmental factors in your discussion.
8. Discussion L161- The results of this study indicated that the highlanders consume adequate DD is 7.7 more likely than lowlanders. But when referring to the inconsistency with a study in South Tigray you have stated it’s because of the mountainous topography, I am afraid to say but the topography of Southern Tigray is not just mountains but a plain and fertile land and the agricultural products in there ranges from crops to fruits. I believe you can cite other possible reason for that the difference such as variety of crops in the areas or the income level or the access to roads and market or education or any other factor? Or you can cite a reference for your justification.
9. Discussion L176- You have mentioned that women possessing radio/mobile are more likely to attain adequate DD and you have related it to high income. This will be a more interesting discussion if you could include the income level (500-1500 birr) you have stated in the result section to support your justification.
10. Discussion L190- You claimed to have focused on the most vulnerable group. Can you please elaborate on this by rewriting it as ‘…the most vulnerable group such as …’ since it is open to interpretations. The vulnerable group can be attributed to age (children, aged), sex, physical and economic condition etc.
11. Conclusion: Your conclusion is weak and it requires writing it very well.

Minor

1. Abstract L41- Please remove the repeated word.. reproductive “…undertaken among 634 reproductive women of reproductive age.” scientific names should be italicized
2. Abstract L48- re write the sentence “Women those who were living…” as Women who were living
3. Background L95- In the sentence “…mothers' lack of resistance to” please either complete the sentence or remove the ‘to’ at the end.
4. Methods L 111- Typo error, change If to if
5. Methods L 1120- I am not sure if this is a standard abbreviation ‘32hh’ or you should write it in full at first as ‘32 household’
6. I have observed that throughout the manuscript you have capitalized the word women ‘Women’. I didn’t find it appropriate please check it.
7. The grammatical problem is quite high, for instance read the lines between L149-L155 in the last paragraph of the result section. In my opinion your manuscript need professional editing to solve the language issues.
