# Supplementary material for: Dietary diversity and associated factors among women of reproductive age in Jeldu District, West Shoa Zone, Oromia Ethiopia
Source: PLoS One. 2022 Dec 19;17(12):e0279223. doi: 10.1371/journal.pone.0279223 (PMC9762567; doi:10.1371/journal.pone.0279223)
Supplement: Attachment — Submitted filename: PLOSE ONE (Comment).docx [file pone.0279223.s003.docx]

**PLOSE ONE: COMMENTS**

**Research title: Dietary diversity and associated factors among reproductive age women in Jeldu District**

| **S.N** | **Section** | **Comments** |
| --- | --- | --- |
| 1 | **Abstract (line 36-37)** | - The authors should include statement that indicates a problem on dietary diversity - Objectives : Well stated - Methods : What does the authors wanted to mean when they say charts were computed (Line 45) - Add also P value to the 95% CI (Line 45) - Results: Make the language clear (48-51) |
| 2 | **Background** | At the end of the background, the authors should clearly indicate gap. They should clearly indicate what has been done before, what should be done and the contribution of the current study |
| 3 | **Methods** | - The authors should clearly describe the sampling technique. The language in this methods section should be revised so that it will be clear (Line 113-115). - It says ‘’Pre-test was conducted on 32 hh ‘’. The hh should be written out throughout as it may not be clear for readers (Line 120) |
| 4 | **Results** | - The result , information should be clearly presented   For instance, the information presented , more than (…….) is incomplete (Line 137), More than half (62.1 %) ….which is not consistent (Line 140)   - A woman’s making decision… For one thing decision on what??? the other issue is the poor language that the authors should improve (line 152) |
| 5 | **Discussions** | - The higher proportion of dietary diversity in study area is due to the nature of study and study period. What does this mean??? What evidence do the authors have?? they have briefly describe (Line 161) |
| 6 | **Conclusion** | - The authors said concerned body should give special attention. This is very vague which the authors should make it targeted |
| 7 | **General comments** | - There are a lot of typographical errors in this manuscript. The language and logic flow of ides should be maintained - If all the comments are incorporated , I belief the article is of great importance in contributing to scientific evidence |
