# Supplementary material for: Dietary diversity and associated factors among women of reproductive age in Jeldu District, West Shoa Zone, Oromia Ethiopia
Source: PLoS One. 2022 Dec 19;17(12):e0279223. doi: 10.1371/journal.pone.0279223 (PMC9762567; doi:10.1371/journal.pone.0279223)
Supplement: Attachment — Submitted filename: PLOS Comments from reviewer.docx [file pone.0279223.s004.docx]

**Reviewer 1**

**Comments to the Author**

The topic for this article is an interesting and important one. It presents information on dietary diversity and associated factors among children aged 6 - 23 months in Ethiopia. This is useful information; however, the reviewers have raised some important issues that deserve attention.

I suggest the authors identify the gap in the current literature a little bit more clearly, even for Ethiopia since a number of studies are already available. Furthermore, the generalizability of the present study to other populations is a question. It should become clear what is the added value of this study beyond Ethiopia, especially considering that the type of study is not very innovative. Likewise, your results could be compared to other parts of the world.The manuscript does not bring any novelty to the field, and it uses incorrect terms, making it difficult to understand the study. Hence justify for the above sentence?

**Comments**

1. In abstract…p value: for binary and multivariate should be sated
2. In abstract-

-Under result, is there highland and highland place in Jaldu district which is stated by CSA Ethiopia? If yes, could u show for us the reference?

Your result in abstract is different from the factor associate Written under line 149-155 ’’- The logistic regression output showed that Women’s making decision, Agro ecological zone having large livestock, having radio and mobile phone significantly associated with dietary diversity women who were living in highland were 7.71 (AOR =7.71; 95% CI: 3.72,15.99) , women who have radio were1.87(AOR= 1.87;95% ;1.17,2.99), Women who making decision were 3.93(AOR =3.93 ; 95% CI: 3.93,2.3,6.71) , women who had large livestock were 2.67( 154 AOR;2.67: 95% CI;1.4,5.08) and women who have mobile phone1.92 (AOR=1.92:95% 155 CI;1.74,3.16,) had adequate dietary diversity (Table 3).’’ Could you justify or correct this thing?

3. Is it reference -1 is book or report or article? Could revise its citation?
4. I think the bracket of PLOS citation according to plos guideline is like= [ ]

**Methods**

5. Change to methods=method and materials….line 100
6. Write subtitle under method and materials like=Study design, setting, and population….etc until quality control
7. How 10 kebeles were selected? And from how many kebeles?
8. Why you want to use 1.5 design effects as fara as u want to generalize your finding for one district or Jaldu district, not west shoa zone.
9. Correct capital to small letter “If” ….line 111

**Result**

10. Break a sentence in to two and make a sense…. from 134-137
11. There is no variables in table 1, which you wrote here under result ‘’More than (53.6%) of the respondents were protestant in religion, about 83% were 138 married, 55.2% participants, were had no formal education. 57.1% respondents were farmers 139 and 50.6% participants living with less than five family member(table.1).”
12. Make a subtitles under result (socio demographic, factors associated….)
13. On table three under age category (35—44) it shows as it was associated (AOR;2.75(1.02,7.4),P= 0.045),Hence why u did not discussed?

Discussion

14. One line 161 ‘’ the high proportion of adequate dietary diversity in this study is due to the nature of the study i.e study period’’. Justify deeply with citation why the prevalence was high?
15. On line 172-176, ’’The difference is due to agro ecological zone, production availability and socio cultural determinants, most of the highland areas are in Tigray Ethiopia are mountainous and prone to soil erosion and degradation; these could be risk factors for a reduction in agricultural productivity when variety of production are not existing an individual cannot consumed varieties of food groups’’, the question here is, justify how your study period got high food diversity at highland than lowland as far as most of the highland areas are in Tigray Ethiopia are mountainous and prone to soil erosion and degradation; these could be risk factors for a reduction in agricultural productivity?
