# Supplementary material for: Dietary diversity and associated factors among women of reproductive age in Jeldu District, West Shoa Zone, Oromia Ethiopia
Source: PLoS One. 2022 Dec 19;17(12):e0279223. doi: 10.1371/journal.pone.0279223 (PMC9762567; doi:10.1371/journal.pone.0279223)
Supplement: Attachment — Submitted filename: Response to reviewers.docx [file pone.0279223.s007.docx]

**Author's response to Reviewers Comments**

PONE-D-22-08826

**Title of Manuscript:** Dietary Diversity and Associated Factors among Reproductive Age Women in Jeldu District, West Shoa Zone, Oromia Ethiopia

**Authors:**

1. Gudisa Merga: [gudisamerga2017@gmail.com](mailto:gudisamerga2017@gmail.com)
2. Samson Mideksa: [samkmwmtj@gmail.com](mailto:samkmwmtj@gmail.com)
3. Nagasa Dida: [nadibefe@yahoo.com](mailto:nadibefe@yahoo.com)

***Generally, in manuscript we have used track change to incorporate or address the reviewers’ comments.***

**A Point by point response**

**Response to Reviewer #1:**

**RESPONSE TO SPECIFIC COMMENTS:**

- Write in full in the first instance it occurs and be sure each occur minimum two times or more in the text, unless use the full text than the abbrevaitions

**Response from the authors:** done has indicated.

- Show the gap why this research is done rather than telling the importance of DD
- Better merge it with the background

**Response from the authors:** the background has revised and merged with the objective as commented by the reviewer.

- How charts will be computed rather charts can be used to present the results, revise

**Response from the authors:** Revised

- Better you modify this term – ‘achieved’, describe it as it appears in your first objective

**Response from the authors:** replaced with ‘consumed’

- Where is the knowledge gap, design or intervention gap this study filled, please include, re-write

**Response from the authors:** revised and re-written.

- Ethics approval and consent to participate: Too shallow please include all the components of ethical conduct of a study

**Response from the authors:** revised and written in detail.

- Please include only the most relevant issues it is too much in a single chart, what are the other fruits

**Response from the authors:** The variables under the chart are the standard food group variables used to assess the dietary diversity. If we reduce the variable it become incomplete and the other fruits also part of it.

- too shallow discussion discuss further additionally relevant findings

**Response from the authors:** We have tried.

- Include strength and limitation of the study.
  - **Response from the authors:** The last paragraph of the discussion is the strength and limitation of the study. It has also elaborated more.
